# Elucidating the Role of *Chmp1* Overexpression in the Transport of Polyamines in *Drosophila melanogaster*

**DOI:** 10.3390/medsci10030045

**Published:** 2022-08-25

**Authors:** Coryn L. Stump, Robert A. Casero, Otto Phanstiel, Justin R. DiAngelo, Shannon L. Nowotarski

**Affiliations:** 1 Division of Science, Pennsylvania State University, Berks Campus, Reading, PA 19610, USA; 2 Sidney Kimmel Comprehensive Cancer Center, Johns Hopkins University, Baltimore, MD 21287, USA; 3 Department of Medical Education, College of Medicine, University of Central Florida, Orlando, FL 32827, USA

**Keywords:** polyamine transport system, *Drosophila melanogaster*, imaginal disc assay, Chmp1

## Abstract

Polyamines are small organic cations that are essential for many biological processes such as cell proliferation and cell cycle progression. While the metabolism of polyamines has been well studied, the mechanisms by which polyamines are transported into and out of cells are poorly understood. Here, we describe a novel role of Chmp1, a vesicular trafficking protein, in the transport of polyamines using a well-defined leg imaginal disc assay in *Drosophila melanogaster* larvae. We show that *Chmp1* overexpression had no effect on leg development in *Drosophila*, but does attenuate the negative impact on leg development of Ant44, a cytotoxic drug known to enter cells through the polyamine transport system (PTS), suggesting that the overexpression of *Chmp1* downregulated the PTS. Moreover, we showed that the addition of spermine did not rescue the leg development in *Chmp1*-overexpressing leg discs treated with difluoromethylornithine (DFMO), an inhibitor of polyamine metabolism, while putrescine and spermidine did, suggesting that there may be unique mechanisms of import for individual polyamines. Thus, our data provide novel insight into the underlying mechanisms that are involved in polyamine transport and highlight the utility of the *Drosophila* imaginal disc assay as a fast and easy way to study potential players involved in the PTS.

## 1. Introduction

The polyamines putrescine, spermidine and spermine are small organic cations that are essential for normal cell growth and development [1,2]. Under normal cellular conditions, polyamine levels are maintained in the millimolar range through biosynthetic, catabolic and poorly characterized transport mechanisms [3,4,5,6,7]. Maintaining homeostatic levels of the polyamines is important, as dysregulation of the polyamines can be associated with physiological conditions such as cancer, aging and Parkinson’s disease [8,9,10].

Polyamines have been shown to enter the cell via a transporter in simple eukaryotes and prokaryotes, and these mechanisms have been well-characterized [5,11]. However, to date, the exact mechanism(s) surrounding a polyamine transporter in higher eukaryotes is unclear. It is known that in higher eukaryotes, polyamine uptake is mediated by an energy-dependent mechanism that is Na^+^-independent but membrane potential-dependent. Moreover, Ca^2+^ or Mg^2+^ is necessary for the activity of the polyamine transport system (PTS). Based on kinetic data, there is evidence to suggest that putrescine enters the cell via its own carrier while spermidine and spermine enter via different carrier(s) [5]. Currently, there are three models for the PTS in higher eukaryotes [12,13,14]. One common feature amongst all three hypothesized models is that polyamines are sequestered in vesicles upon entry into the cell.

In fitting with the theory that polyamines are sequestered in vesicles upon entry into the cell, preliminary experiments in our laboratory identified a number of genes that function in the endomembrane system that are differentially expressed in H157R cells. These H157R cells are deficient in the PTS [15]. One such protein, charged multivesicular body protein 1a (Chmp1A), is involved in vesicular trafficking and is the focus of these studies. Chmp1A is a component of the endosomal sorting complex required for transport-III (ESCRT-III) [16,17]. Proteins and other molecules that enter the cell and move through the endomembrane system must do so in membrane-bound compartments known as endosomes. Endosomes can be used to recycle transmembrane receptors that have been endocytosed from the plasma membrane, transport proteins traveling between compartments such as the plasma membrane and the golgi network, or transport proteins that are targeted for destruction in the lysosome. Many different classes of proteins function to generate vesicles in the endomembrane system and to sort proteins to the appropriate target compartments, including the Rab and SNARE proteins. In addition, ESCRT proteins are important for sorting proteins in the multivesicular body, or maturing endosome, and sorting nexin proteins are important for sorting transmembrane proteins back to the plasma membrane from recycling endosomes [16,18]. However, whether these vesicular trafficking proteins function in regulating polyamine transport is not known.

*Drosophila melanogaster (D. melanogaster* or *Drosophila*), commonly known as the fruit fly, is emerging as a model organism for studying the polyamine pathway due to the conservation of many of the enzymes involved in polyamine metabolism and catabolism between flies and humans [19,20,21]. Recently, an elegant system was developed using the *Drosophila* third instar larval imaginal leg disc to study polyamine transport [22,23]. Combining this leg imaginal disc system and the ease of genetic manipulations in *Drosophila* makes the fly an ideal system to study the genes important for controlling polyamine transport [24].

In these studies, the imaginal leg disc assay in *Drosophila* was used to characterize the impact of *Chmp1* (the *Drosophila* homolog to Chmp1A) overexpression on the PTS. Here, we demonstrate that *Chmp1* plays a role in the PTS, specifically by downregulating spermine import. Further, our data suggest the existence of multiple polyamine transporters in *Drosophila* and may better our understanding of the PTS in other eukaryotes.

## 2. Materials and Methods

### 2.1. Cell Lines

Human lung carcinoma NCI H157G and H157R cells were cultured in RPMI 1640 media (ThermoFisher, Leesport, PA, USA) supplemented with 10% bovine calf serum (Atlanta Biologicals, Flowery Branch, GA, USA) and 0.4 mg/mL Geneticin (G418) (ThermoFisher) [15]. Stock flasks were incubated at 37 °C in a humidified atmosphere of 95% air and 5% CO_2_.

### 2.2. Western Blot Analysis

Western blotting was conducted as previously described [4]. The antibodies recognizing Chmp1A (Sigma, St. Louis, MO, USA) and Actin (Santa Cruz, Dallas, TX, USA) were used in a 1:1000 dilution. The fluorescent secondary antibodies were detected by infrared using the Odyssey imaging system (Li-Cor Biosciences, Lincoln, NE, USA).

### 2.3. Fly Genetics

Flies used in this study include: *w* [1118]*; P{w[+mW.hs] = GawB}MJ33a* (BL#6992); w[*]; P{w[+mC] = UAS − GFP.S65T}Myo31DF[T2] (BL#1521); *UAS-Chmp1; D* [1]*/TM3Ser* [25]. Flies were grown on cornmeal–sugar–yeast medium (9 g *Drosophila* agar (Genesee Scientific, El Cajon, CA, USA), 100 mL Karo Lite Corn Syrup, 65 g cornmeal, 40 g sucrose, and 25 g whole yeast in 1.25 L water) on a 12 h:12 h light:dark cycle at 25 °C. Additional whole yeast was sprinkled onto the food of the experimental crosses and imaginal leg discs were dissected from wandering 3rd instar larvae.

### 2.4. Imaginal Disc Assay

Leg imaginal disc assays were performed as previously described [22]. For our experiments, discs were incubated in a solution of 1X minimal Robb’s medium [22] for 18 h at 25 °C. Various treatment groups were studied in our leg imaginal disc assays using the following final concentrations: 20-hydroxyecdysone (hydroxy) (1 µg/mL), Ant 44 (50 µM), difluoromethylornithine (DFMO) (10 mM), putrescine (200 µM), spermidine (200 µM) and spermine (200 µM). After 18 h of incubation under the treatment conditions, the discs were scored as developed or not developed. Fully developed discs in which the leg protrudes from the epithelium and partially developed discs in which the leg protrudes from the epithelium but is not fully extended, were scored as developed. Not developed discs exhibited no development. For each experiment, the percent development was determined for the treatment group and was considered as [(number of developed discs for that treatment group)/(total number of discs for that treatment group)] × 100.

### 2.5. Statistical Analysis

The results were expressed as mean ± standard error (SE). Comparisons between experimental (Chmp1-overexpressing) and control (GFP-overexpressing) conditions were made using unpaired Student’s *t*-tests. *p* < 0.05 was considered statistically significant.

## 3. Results

### 3.1. Chmp1A Is Overexpressed in H157 Cells That Are Resistant to MGBG

**Figure 1:**
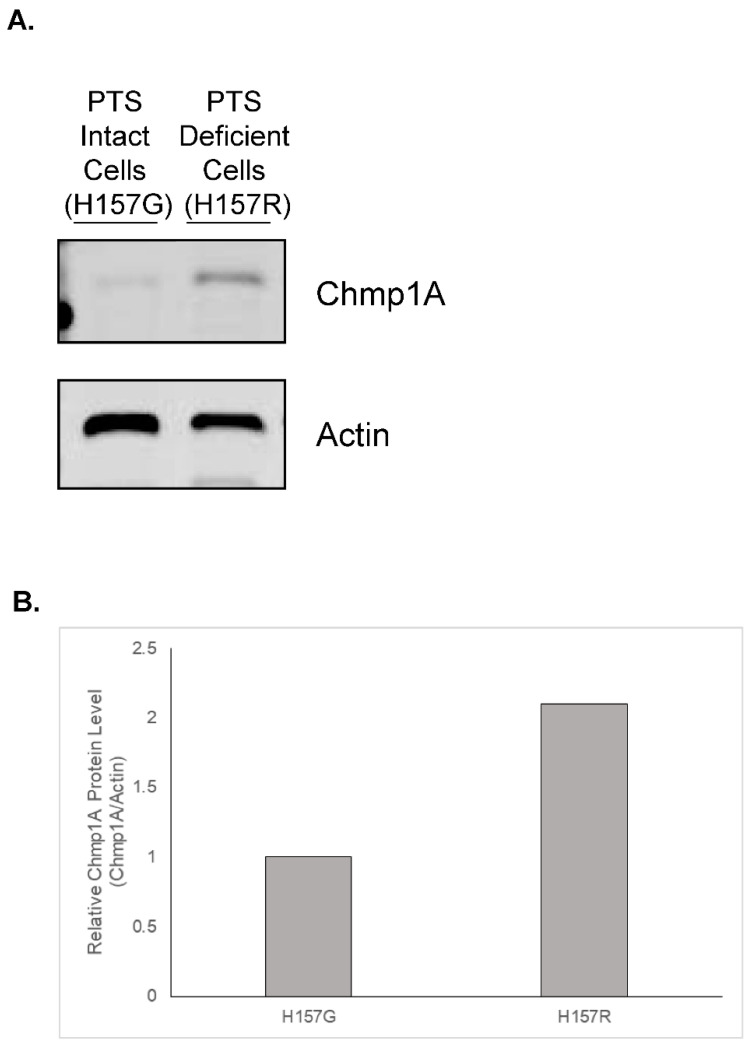
Chmp1A protein is increased in PTS-deficient H157 cells when compared to PTS intact H157 cells. Total protein from NCI H157 cells with intact PTS (denoted as H157G) or deficient in the PTS (denoted as H157R) was harvested. Chmp1A protein levels were assessed via Western blot analysis and Actin was used to normalize protein levels. This was done in duplicate with reproducible results. (**A**) A representative image is shown. The original western blots were provided as Figure S1. (**B**) Western blot quantitation. The relative Chmp1A protein density was normalized to Actin and is shown as fold change when compared to H157G cells.

Various cell types have been identified that have a defect in transporting polyamines into cells [26,27,28]. A lineage of the lung carcinoma cell line, NCI H157, underwent insertional mutagenesis and the cells, denoted as H157R, were found to be resistant to the cytotoxic effect of methylglyoxal bis(guanylhydrazone) (MGBG), a drug that enters the cell through the PTS [15]. Interestingly, these cells were also unable to transport exogenous polyamines [15]. Charged multivesicular body protein 1a (Chmp1A) was shown to be upregulated in these cells when compared to H157 cells that had an intact PTS (Figure 1). Consistent with the PTS hypotheses described above, the data suggest that proteins involved in endosomal sorting may play a role in the regulation of intracellular polyamines once they are brought into the cell. However, genetically manipulating H157R cells to further test the role of Chmp1A in PTS proved to be challenging; therefore, we decided to take advantage of the power of *Drosophila* genetics and the simplicity of the fly system to further define the role of *Chmp1A* in regulating the PTS. *Drosophila* have a single *Chmp1A* gene (denoted as *Chmp1*), while humans have two. This allows for easier genetic manipulation of the *Chmp1* gene in flies. In fact, overexpressing constructs have already been generated and characterized [25]. Moreover, using the well-established imaginal disc assay provided an excellent system to study if *Chmp1* was involved in the import of polyamines [22].

### 3.2. The Overexpression of the Drosophila Chmp1 Gene Blocks Ant44 Transport in Leg Imaginal Discs

**Figure 2:**
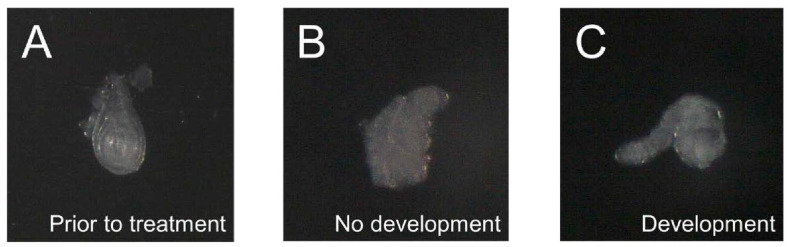
Leg imaginal discs dissected from *Chmp1*-overexpressing *Drosophila melanogaster* do develop in the presence of 20-hydroxyecdysone. (**A**) Depiction of a representative leg imaginal disc that was dissected from *MJ33a-Gal4 > Chmp1* (*Chmp1*-overexpressing) wandering 3rd instar larvae immediately following dissection, (**B**) after 18 h at 25 °C in the absence of 20-hydroxyecdysone showing a disc that is not developed, and (**C**) after 18 h at 25 °C in the presence of 20-hydroxyecdysone showing a disc that is developed.

**Figure 3:**
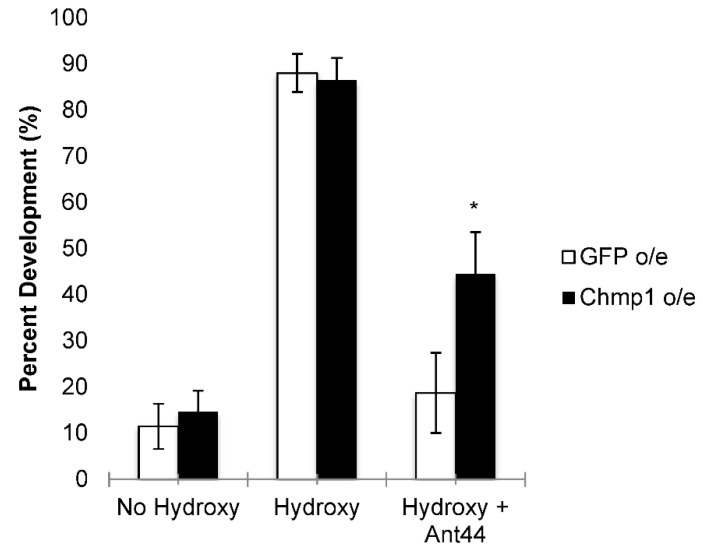
*Chmp1* overexpression blunts the developmental effects of Ant44 on leg development. Leg imaginal discs were dissected from *MJ33a-Gal4 > GFP* (*GFP*-overexpressing, denoted GFP o/e) and *MJ33a-Gal4 > Chmp1* (*Chmp1*-overexpressing, denoted Chmp1 o/e) wandering 3rd instar larvae. Discs were incubated for 18 h at 25 °C in the presence or absence of 20-hydroxyecdysone (hydroxy) and in the presence or absence of 50 µM Ant44 (Ant44). The development of discs was scored and the percentage of disc development was recorded for each experiment. The data are shown as the mean ± SEM (*n* = 16–34). * *p* < 0.05 using a student’s *t*-test comparing GFP o/e to Chmp1 o/e.

*Chmp1* was overexpressed in imaginal leg discs using *MJ33a-Gal4*, a Gal4 line previously shown to express ubiquitously in leg imaginal discs, and compared to *GFP*-overexpressing controls [29]. Imaginal discs were dissected from *GFP*- and *Chmp1*-overexpressing larvae and incubated with 20-hydroxyecdysone (Figure 2) [22]. The overexpression of *Chmp1* was not lethal as the imaginal discs developed normally when in the presence of 20-hydroxyecdysone. The percent development of the imaginal discs in the *Chmp1*-overexpressing larvae was the same as the *GFP*-overexpressing control larvae suggesting that the overexpression of *Chmp1* did not alter leg development under normal conditions (Figure 3). The development of harvested leg imaginal discs was then assessed in the presence of Ant44, a drug known to enter the cell via the PTS and prevent leg imaginal disc development (Figure 3) [22,23]. Interestingly, larvae that overexpress *Chmp1* showed a 2-fold increase in leg development when compared to the controls. These data suggest that *Chmp1* overexpression inhibits the uptake of Ant44, a cytotoxic compound known to enter the cell via the PTS, implying that *Chmp1* is involved in the PTS in *Drosophila*.

### 3.3. The Overexpression of Drosophila Chmp1 Inhibits the Import of Spermine in DFMO-Treated Imaginal Discs

**Figure 4:**
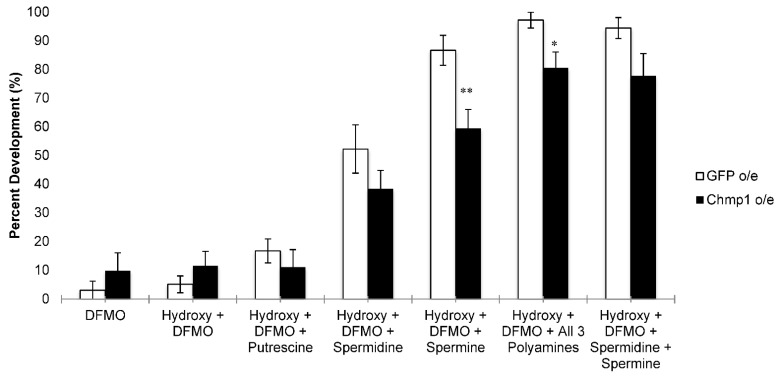
*Chmp1* overexpression alters the ability of polyamines to rescue imaginal disc development in the presence of DFMO. Leg imaginal discs were dissected from *MJ33a-Gal4 > GFP* (*GFP*-overexpressing, denoted GFP o/e) and *MJ33a-Gal4 > Chmp1* (*Chmp1*-overexpressing, denoted Chmp1 o/e) larvae. Discs were incubated for 18 h at 25 °C in the presence or absence of 20-hydroxyecdysone (hydroxy), 10 mM DFMO, and either 200 µM putrescine, 200 µM spermidine, 200 µM spermine, 200 µM all three polyamines or 200 µM spermidine and 200 µM spermine. The development of discs was scored and the percentage of disc development was recorded for each experiment. The data are shown as the mean ± SEM (*n* = 9–26). * *p* < 0.05; ** *p* < 0.01 using a student’s *t*-test comparing GFP o/e to Chmp1 o/e.

In order to determine whether the overexpression of *Chmp1* affects the import of the natural polyamines, polyamine rescue experiments were performed in the presence of both 20-hydroxyecdysone and DFMO. DFMO is a cytostatic compound that inhibits the synthesis of polyamines [30]. Specifically, DFMO inhibits the activity of ornithine decarboxylase (ODC) which converts ornithine into putrescine (a rate-determining step in polyamine biosynthesis). When cells are treated with DFMO, the intracellular polyamines are depleted and the cell relies on the import of exogenous polyamines to survive. Imaginal discs from both the *Chmp1*-overexpressing larvae and the controls did not develop into legs when incubated with DFMO and 20-hydroxyecdysone (Figure 4). However, when imaginal discs were also incubated with putrescine, a modest increase in leg development was seen in the controls while the *Chmp1*-overexpressing cells exhibited no change in the percentage of leg development. These data suggest that putrescine is able to enter the cell and limit the effect of DFMO on leg development in *Chmp1*-overexpressing and control animals (Figure 4). Similarly, incubating imaginal discs with spermidine led to an increase in leg development in both the controls and *Chmp1*-overexpressing larvae (Figure 4). Interestingly, when imaginal discs were incubated with spermine, the control larvae displayed significantly more leg development than the *Chmp1*-overexpressing larvae suggesting that the overexpression of *Chmp1* blunts the transport of spermine into the cell (Figure 4). This was also seen when imaginal discs were incubated with all 3 polyamines. *Chmp1*-overexpressing discs that were incubated with only the higher polyamines spermidine and spermine trended towards fewer developed legs when compared to control larvae (*p*-value = 0.07) (Figure 4). Together, these data suggest that in the fly model system there may be multiple transporters for the polyamines.

## 4. Discussion

In these studies, we show for the first time that *Chmp1* is involved in the PTS in *Drosophila*. We show that the overexpression of *Chmp1* corresponds with a decreased sensitivity to Ant44, a compound known to enter cells via the PTS [22,23] (Figure 3). Interestingly, we also show that the overexpression of *Chmp1* specifically alters the ability of spermine to rescue imaginal disc development in the presence of DFMO, suggesting that the import of spermine may, in part, be via a different mechanism in these animals and may be dependent on *Chmp1* expression (Figure 4). Moreover, the data from *Drosophila* align with studies from the human H157 cell line in which cells that overexpress *Chmp1A* exhibit a defect in transporting the polyamines; thus, further suggesting that this may be a player involved in the PTS (Figure 1) [15].

These data support the results of previous studies that have also utilized the fly leg imaginal disc assay. Wang et al. have previously shown that spermine was better at blocking the inhibition of disc development by Ant44 [22]. Similarly, in our studies, we have shown that spermine was less able to rescue the inhibitory effects of DFMO in *Chmp1*-overexpressing larvae. Combined, these data suggest that in the fly the mechanism of spermine transport may be different than the transport of putrescine and spermidine and support the hypothesis that there are multiple transporters of the polyamines in flies.

The transporter(s) of the polyamines remains enigmatic in both flies and humans. However, the mechanisms surrounding the import and export of polyamines is continually being characterized. In fact, recently it has been shown that two ATPases (132A2 and 132A3) are involved in polyamine transport in humans [31,32]. One hypothesis is that polyamines can be stored in polyamine-sequestering vesicles (PSVs) [5]. A vesicular transporter for spermidine and spermine was described using crude synaptic vesicle fractions from the rat [33]. More recently, SLC18B1 was identified as a vesicular polyamine transporter [34]. In fitting with the hypothesis that the import and export of polyamines may be more complex and involve vesicular trafficking, we hypothesized that the overexpression of *Chmp1* generated additional PSVs from the multivesicular bodies to sequester Ant44 and/or polyamines [35]. In accordance with our data, we propose that the overexpression of *Chmp1* aids in the decrease of polyamines, namely spermine, by sequestering the unidentified transporter of spermine in compartments of the late endocytic pathway. This would lead to less spermine being transported into the cell and could explain the data we observed in our polyamine rescue experiments. However, this mechanism of import needs to be further elucidated in both fly and humans and the transporter(s) need to be identified in order to fully understand the transport of polyamines.

Finally, our studies reaffirm the value of utilizing the *Drosophila* imaginal disc assay as an easy and inexpensive way to study novel players involved in the PTS. In recent years flies have become an important model system for studying polyamine metabolism and transport [19,22,23,36]. This is due to the high homology of polyamine enzymes between the fly and humans and the ease of genetic manipulations that are often challenging to do in tissue culture and mammalian model systems. These studies highlight a novel player, *Chmp1*, involved in the transport of polyamines in flies, and potentially humans. These studies attest to the complexity of polyamine import and export in higher eukaryotes and substantiate the need for more research into the PTS.

## Supplementary Materials

The following supporting information can be downloaded at: https://www.mdpi.com/article/10.3390/medsci10030045/s1, Figure S1: original western blot.
