# Supplementary figures and images for: Elucidating the Role of Chmp1 Overexpression in the Transport of Polyamines in Drosophila melanogaster
Source: Med Sci (Basel). 2022 Aug 25;10(3):45. doi: 10.3390/medsci10030045 (PMC9502369; doi:10.3390/medsci10030045)

Figure S1. Original Western Blot.

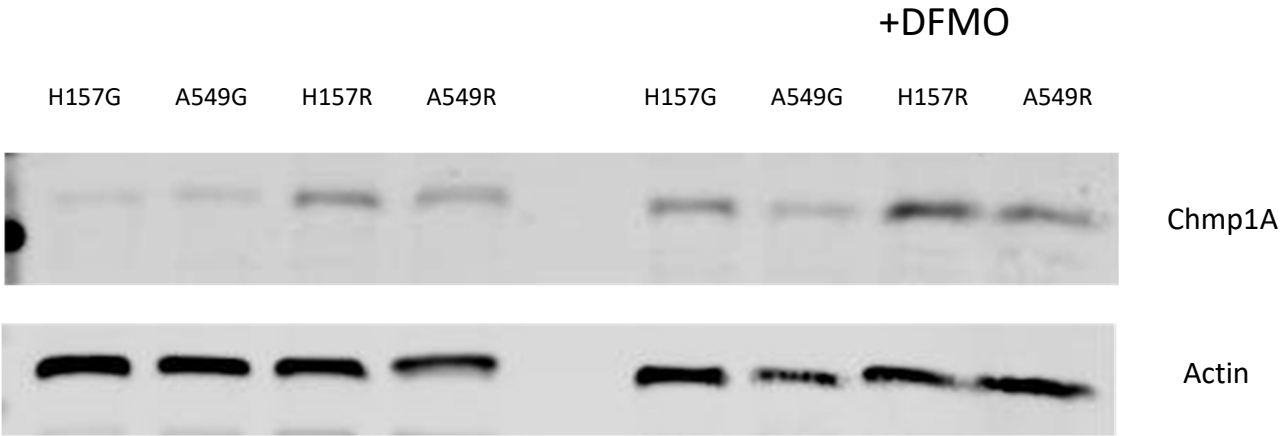

Supplement: Supplementary file 1 [file medsci-10-00045-s001.zip › medsci-1852760 SM.pdf]
